# Headache prevalence and characteristics among adolescents in the general population: a comparison between retrospect questionnaire and prospective paper diary data

**DOI:** 10.1186/1129-2377-15-80

**Published:** 2014-11-27

**Authors:** Bo Larsson, Åsa Fichtel

**Affiliations:** 1 Regional Centre for Child and Youth Mental Health and Child Welfare, Faculty of Medicine, Norwegian University of Science and Technology, Trondheim, Norway; 2 European Union Strategist, Coordination Association of Uppsala County, Uppsala S-73375, Sweden; 3 Department of Public Health and Caring Sciences, Uppsala University, Uppsala, Sweden

**Keywords:** Recurrent headache, Epidemiology, Assessment, Diary, School, Adolescents

## Abstract

**Background:**

In the present school-based study, a convenience sample of 237 adolescents in grade 6-9 and second year in high school (age 12-18 years) was recruited from a city and a smaller town. The aim of the study was to compare information on the prevalence and various characteristics of headaches not related to disease in a retrospect questionnaire and prospective daily recordings of headaches in a standard paper diary during a 3-week period.

**Methods:**

Besides headache severity, number of headache days, intensity levels and duration of headache episodes were estimated with both assessment methods. Most of the school children suffered from tension-type headaches and a smaller portion of migraine attacks.

**Results:**

The overall results showed that school children significantly (p < 0.001) overestimated headache intensity in questionnaires as compared to diary recordings, whereas they underestimated frequency (p < 0.001) and duration (p < 0.001) of headaches. While the correlations on headache severity, frequency and duration between retrospect information in questionnaires and prospective diary recordings were low, the agreement varied with levels of headache characteristics.

**Conclusions:**

Our findings concur well with results from a few similar community studies on headache complaints in school-aged children. We recommend that prospective recordings in diaries should be systematically used in clinical practice but also in epidemiological surveys to increase the validity and reliability in estimates of point prevalence of headache complaints in children and adolescents.

## Background

Headaches are one of the most commonly reported health complaints in school children [1] and the most frequently reported pain in these age groups [2,3]. Numerous epidemiological surveys of headaches in children and adolescents have been conducted over the last decades in various countries and societies [4,5]. The overall mean prevalence of headaches in children and adolescents has been estimated to 54.4-58.4% over periods of 1 month and lifetime [4,5]. Prevalence rates in school-aged children have been reported to be 23-51% for monthly headaches, 6 to 44% for weekly headaches, and 1-9% for daily or almost daily headaches [6,7]. Surveys conducted in Scandinavia, Holland and Taiwan also indicate that school-aged children report frequent headaches with increasing prevalence rates during the last decades [3,4]. Furthermore, frequent headaches in children and adolescents have been associated with negative psychosocial impact such as school absence, lower levels of quality of life, and higher levels of emotional problems, in particular anxiety and depression [8,9] as well as other somatic complaints.

In previous epidemiological surveys and clinical studies, estimation of headaches in children and adolescents has been carried out by means of different assessment methods, the use of various informant sources and sample types. In comparisons between informant sources, the sole use of parent information has been found to substantially underestimate the prevalence of headaches in school-aged children [4,10,11]. Estimates of headaches in these age groups have also been influenced by time frame (lifetime, last year, 6 or 3 months, or the report of weekly headaches) [4,7], differences in item phrasing (“do you suffer from bothersome or have frequent headaches”?). Further, information on headache prevalence has been most commonly collected by means of questionnaires and less often in interviews [4,12].

Although a substantial increase in our present knowledge on headache occurrence in school-aged children has been achieved since Bille’s pioneering study in the 1950s [12], it should be noted that almost all existing information from previous epidemiological surveys on various characteristics of headaches in these age groups has been based on retrospect information susceptible to various degrees of recall bias and error in estimates.

The use of prospective recording of headaches over shorter and longer time periods is likely to reduce such a bias and produce more reliable and valid estimates than global single retrospect reports of varying intervals. In a few previous studies of frequent headaches in children and adolescents in the general population and in clinical outpatient samples, recordings in diaries have typically covered a 2-4 week period [11,13-17]. Longer recording periods from one to 7 months have been used for estimation of migraine attacks [18,19].

Overall, in the studies of children and adolescents with recurrent headaches, comparisons between retrospective information primarily based on questionnaire data versus prospective measurement in a diary, have included clinical samples, or selected individuals with frequent headaches from the general population or school-based samples.

In earlier clinical studies of children primarily suffering from migraine [20-22], information obtained from questionnaires has been found to both overestimate and underestimate frequency and intensity of headache in children and adolescents as compared to prospective diary recordings. In a recent clinical study of adolescents aged 10-18 years with frequent migraine, their recall in a questionnaire showed high agreement with information on headache frequency elicited from an internet-based diary for both 30-day and 90-day intervals [19]. By contrast, in a clinical study of chronic pain including headaches, children and adolescents reported lower pain intensity levels in a diary than in a questionnaire [17].

In a few comparative studies of children and adolescents in community samples, the agreement has been found to be low between retrospective information in questionnaires and interviews compared to prospective recording in diaries on estimates of headache prevalence and various characteristics such as severity, frequency, intensity and duration of episodes [11,13,14].

Given the paucity of systematic comparisons in estimates between retrospect questionnaire and prospective diary data on headaches among adolescents in community samples and degree of discrepancies in ratings, the purposes of the present study of a convenience sample of school children aged 12-18 years recruited from two locations (small town and city) were: (1) to compare information on various headache characteristics such as headache severity, frequency, and duration of headache episodes based on retrospective information in a questionnaire and prospective 3-week recordings in a standardized paper diary and, (2) variation related to headache history, headache type and current headache by gender, grade, and school location.

## Methods

### Sample

**Figure 1:**
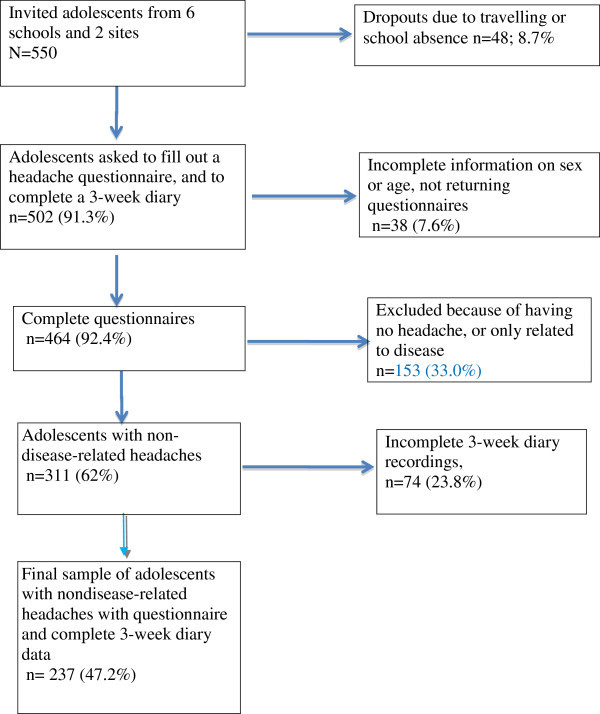
Participant flow in the study.

The present convenience sample of adolescents aged 12-18 years in a regular public school population was recruited from one city (Uppsala with about 187.000 inhabitants, 2007) and one town (Östhammar with about 21000 inhabitants in the municipality, 2007) in the same county in Sweden. In the city, the adolescents were recruited from 5 different schools consisting of four compulsory schools (grade 6-9) and one high school (11th year of schooling). In the town, one compulsory school participated (grade 6-9) and two classes from each grade were invited to participate in the study. The school principals at each school approved the study and selected the classes together with the teachers in the study. The selection process of adolescents in the sample is presented in Figure 1. Out of a total of 550 invited school children, 8.7% (n = 48) were unable to participate due to school absence or travelling. Thus, 502 adolescents (91.3%) were asked to fill out a questionnaire on headache characteristics and to keep a headache diary on paper for three weeks (see details below). Demographic information on gender and age was complete for 464 students (92.4%) and incomplete for 25 students (5%), who returned the questionnaires. Reasons for nonresponse were school absence, illness and unwillingness to participate further in the study. In the questionnaire, adolescents were initially asked whether they had experienced headache during the last year. Those with no headache or only associated with infections, fever or other diseases (n = 163; 35.1%) were asked to stop filling out the form after the initial question.

Of the 502 adolescents, 86.3% of the students (n = 433; 49.9% boys) completed the diaries for the first week, for the second week 81.3% (n = 408; 51% boys), and for the third week 76.7% (n = 385; 51.4% boys). Three complete weeks of diary recordings were performed by 68.1% (n = 342) of the students.

In the final sample of adolescents used for comparisons between questionnaire and diary data, only those with nondisease-related headaches as stated in the questionnaire, and those with complete 3-week headache recordings were included in the analyses (N = 237; 47.2% of the eligible sample). Analysis of relationships between dropout for gender, grade and school location showed significant associations for grade, χ2 (4) = 41.12, p < 0.001, with higher proportions of the youngest and the oldest adolescents completing a 3-week period (93.2% vs 92.7%, respectively) than those in the middle grades (56.1% to 78.5%). In addition, adolescents in the city completed 3-week recordings more often than those in the town (82-1% vs 64.7%, respectively), χ2 (1) = 12.10, p < 0.001. However, no relationship was found between gender and dropout.

### Questionnaire

Adolescents who endorsed having headaches not related to disease during the last year were further asked questions about different headache symptoms covering the ICHD2 criteria and characteristics of headaches [14,23,24]. They were also asked about having two different types of headaches and to complete these symptoms for both types. The questionnaire was previously tested in two samples of schoolchildren and interviews were held with 35 children [23]. The agreement between questionnaires and interviews for the diagnoses of migraine, tension-type headache and unclassified headache was good (85.3%), as was the agreement between interview diagnoses (IHS) and diagnoses (intuitive) set by clinicians on the interview responses (88.6%).

The information included *number of previous headache episodes* as used in the IHS [24] definitions of migraine and tension-type headaches (1-4 times, 5-9 times, 10 times or more), *headache history* (number of years and months), *frequency* (“less than once a month, 1-3 times a month, 1-3 times per week, every day or almost every day”), *severity* of headaches (“How bothersome has your headache been lately?”) rated on 0-5 scale (for details, see Table 1). These ratings were only performed by adolescents in three of the compulsory schools in the town and city (n = 362). The raw scores were recoded into the following three severity levels: mild headaches (a score of 1-2), moderate headaches (a score of 3), and severe headaches (a score of 4-5); *Intensity* levels (“How intense is your headache usually?”) were assessed on the 0-3 scale as recommended by the IHS Clinical Trials Subcommittee [25] (1 = mild, 2 = moderate and 3 = severe headaches; duration on a 1-6 scale (less than 30 minutes, 30 min-1 hour, 1-2 hours, 2-4 hours, 4-12 hours, more than 24 hours), as well as experience of *current headaches* rated on a 0-10 cm visual-analogue scales (VAS) (no data available for the oldest students in high school).

**Table 1:** Medians (IQR) for headache severity, frequency, intensity and duration in the questionnaire and the diary recordings, and means (SDs) for current headaches on the VAS and number of headache days as reported by the adolescents (N = 237)

**Questionnaire**	
Severity^a^	2.0 (1.0)
Frequency^b^	3.0 (1.0)
Intensity^c^	2.0 (1.0)
Duration^d^	2.0 (2.0)
Current headaches^e^	1.91 (2.39)
(Mean/SD)	
**Diary**	
Severity^a^	1.63 (0.75)
Number of headaches per month^f^	12.71 (8.27)
(Mean/SD)	
Frequency^b^	2.0 (1.0)
Duration^g^	1.49 (0.76)

Note. ^a^Severity rated on a 1-5 scale (see details in Table 1); ^b^Frequency on a 1-3 scale.

^c^Intensity on a 1-3 scale; ^d^Duration on a 1-6 scale; ^e^Current headaches rated on a 0-10 visual-analogue scale (VAS); ^f^Frequency rated as number of headache days per month (0-30).

^g^Duration on a 1-4 scale.

SD: Standard deviation; IQR: Interquartile range.

### Headache diary

The paper diary developed by Budzynski and collaborators [26] and revised by Epstein and Adler [27] was chosen because of its extensive use in previous controlled outcome studies on adults with recurrent headaches [28] as well as in our own controlled trials of the effects of school-based treatments for adolescents aged 10 to 18 years [29] and by others [13,17]. The diary has been socially validated against ratings by significant others in adults [26] treated because of recurrent headaches as well as for adolescents [30]. For children with recurrent migraine attacks and adults with frequent migraine or tension-type headaches, a 3-4 week recording period has been suggested to be optimal in regard to compliance, validity and reliability of ratings as well as accuracy of headache frequency assessment compared to longer recording periods [19,31].

In the present study, the adolescents were asked to record their severity of headaches four times a day at approximately 5 hour intervals (at breakfast-7 am, at lunch-12 am, in the afternoon-17 pm, and at bedtime-22 pm) on the following 0-5 intensity scale: 0 = “No headache”; 1 = Very mild headache, only noticeable when attending to it”; 2 = “Mild headache, could be ignored at times”; 3 = “Moderate headache, normal activities can be continued”; 4 = “Severe headache, difficult to concentrate, can manage undemanding tasks”; and 5 = “Extremely intense headache, incapacitated, can’t do anything”.

From the weekly diary, the following measures were extracted: *Headache severity* = mean of ratings of headache episodes using the same scale as in the questionnaire with a score range of 1-5. The raw scores in the questionnaire were recoded into the following three severity levels: low (a score of 1-2), medium (a score of 3), and high (a score of 4-5). *Frequency of headaches* during the diary recording period was assessed as number of headache days = count days when any headache activity was recorded during the day in the diary; *Headache duration* = mean length of headache episodes based on the number of consecutively reports at the four time points of any headache activity in a given day (range: 0-4 with approximately 4 hour intervals).

Information about the study was distributed by the classroom teacher to students and parents who were asked to inform the teacher if the child was not allowed to participate. Participating students returned informed written consent to the classroom teacher. A list with code number coordinating student questionnaires and diaries (not accessible for the research assistant) was kept by the teacher. Research assistants visited each classroom weekly to collect the diaries and also reminded teachers and participants to continue the headache recordings for the following week. The study was conducted in the middle of the spring semester and each class was paid 1500 Swedish crowns (about 200 US dollars) after data collection was terminated.

The study conformed to the revised ethical principles of the Helsinki declaration and the Codex rules and guidelines for research [32,33].

### Statistics

Descriptive statistics included means, standard deviations (SD), medians and interquartile range (IQR), and percentages. Chi-square test was used to examine associations between categorical variables, while Spearman rank-order correlations, r_s_, were used for ordinal variables. In the analysis of agreement between categorical variables, Kappa coefficient was used. Missing values in the diaries were imputed for each individual if less than 15% of their measurement points across the 3-week recordings were omitted by using the expectation maximization (EM) procedure [34]. Differences between independent group means were examined by means of Student t-test or ANOVA using age, gender and location (town vs city) and their interactions as between-group factors. When significant main effects were found, subsequent post hoc testing was performed with Bonferroni contrasts. For within-group and paired measurement on ordinal scales, the Wilcoxon signed ranks test was used. A p-value of 0.05 or less indicated statistical significance for two-tailed tests and SPSS 20.0 was used in the analyses.

## Results

### No or disease-related headaches

About one third (35.1%, n = 163) of school adolescents reported having no headaches or headaches only occurring when having fever, cold or related to other disease. Boys (21.5%) reported no or such headaches less often than girls (44.7%), χ2 (1) = 28.90, p < 0.001. Students in the highest grade had the lowest proportion of no headache or disease-related headaches (12.5%) as compared to those in the lower grades (39.5%), χ2 (4) = 16.98, p < 0.01, and students in the town had higher proportion of no headache or disease-related headaches (43.6%) than those in the city (35.0%), an association approaching significance, χ2 (1) = 3.53, p = 0.06 when controlled for grade.

### Headaches not related to disease

In the following, results are presented only for adolescents who in the questionnaire reported nondisease-related headaches, and also completed prospective 3-week recordings in the diary (N = 237) (see Figure 1).

#### Headache history

The students who provided information on headache history (n = 132; 55.7%) had had recurrent headaches for a mean of 4.5 years (SD 1.92), and only two of them reported having had headaches for about a year, all the others for longer periods. Of the headache sufferers, 26.5% had had headaches for >1-3 years, 58.3% for 4-6 years, and 15.2% for a period of 7 years or longer. There was no difference in headache history related to gender or school location.

#### Number of previous headache episodes

Of the adolescents, 16.4% had previously experienced either 1-4 or 5-9 headache episodes, and about two thirds (67.1%) had had at least 10 previous episodes with no difference between gender or school location. As expected, students in the two lower grades had experienced significantly lower number of headache episodes than those in the upper grades, χ2 (8) = 17.83, p < 0.05.

#### Headache type

Using the ICHD2 criteria for establishing headache diagnosis in the questionnaire, the distribution of headache types is shown in Table 2. We found no significant association between headache type (migraine, tension-type, migraine and tension-type headaches combined and unspecified headaches) by gender, grade and school location.

**Table 2:** Characteristics of the final sample of adolescents (N = 237) with figures in numbers and percentages (%)

**Gender**	
Boys	102 (43%)
Girls	135 (57%)
**Grade**	
6 (11-13 years)	69 (29.1%)
7-8 (13-15 years)	68 (27.8%)
9 (15-16 years)	63 (26.6%)
11 (2^nd^ grade high school)	39 (16.5%)
(17-18 years)	
**Location**	
City	161 (67.9%)
Town	76 (32.1%)
**Headache diagnosis**	
Migraine	37 (15.6%)
Probable migraine	22 (9.3%)
Tension-type headache	39 (16.5%)
Probable tension-type	50 (21.1%)
Probable migraine & tension-type headache	15 (6.3%)
Unspecified headache	74 (31.2%)

### Associations between questionnaire and diary data

Means (SDs) and medians (IQRs) for the various headache parameters in the questionnaire and diary recordings are presented in Table 3. Relationships between the two information methods were examined for estimates of headache severity, frequency, here number of headache days subgrouped into three categories, and duration. The findings on the VAS for current headaches and various intensity levels were also compared with other characteristics in the questionnaire and diary recordings.

**Table 3:** Spearman rho correlations for questionnaire and 3-week diary data on headache severity, frequency, intensity, duration levels and current headaches as reported by adolescents in questionnaire and paper diary

	**Questionnaire**					**Diary**		
**Measure**	**Severity**^**a **^**(scale 1-4)**	**Frequency (scale 1-5)**	**Intensity**^**b **^**(scale 1-3)**	**Duration (scale 1-6)**	**Current headache (VAS 0-10)**	**Severity/**^**a **^**episode (scale 1-5)**	**Number of headache days/mo (scale 0-30)**	**Duration/episode (scale 1-4)**
Severity^a^	——							
Frequency	−0.31***	——						
Intensity^b^	0.51***	−0.10	——					
Duration	0.35***	0.10	0.37***	——				
Current headache	0.13	−0.33***	0.14	−0.06	——			
Severity/e	0.32***	0.09	0.24*’	0.18**	0.04	——		
Number of headache days	0.14	−0.11	0.19**	0.02	0.34***	0.00	——	
Duration/e	0.20*	0.02	0.24**	0.31***	0.12	0.25***	0.38***	——

Note. *p < 0.05; **p < 0.01; ***p < 0.001. Sample size for correlations varied from 120 to 231.

^a^Descriptive numeric 1-5 scale; ^b^Intensity levels: Mild, Moderate and Severe. VAS: Visuo-analogue scale.

#### Headache severity

In the questionnaire ratings on the descriptive, numeric 1-5 scale about half of the adolescents (55.0%) reported low severity levels of headaches (a score of 1-2), about a quarter had medium levels (26.7%) (a score of 3), and one fifth (18.3%) high levels of headaches (a score of 4-5). Girls reported high severity levels more often than boys (25.7% vs 8.8%, respectively), a significant association, χ2 (2) = 6.32, p < 0.05. While the association to grade was nonsignificant, a significantly higher proportion of students in the town had high levels of headaches (23.0%) than those in the city (12.3%), χ2 (2) = 7.41, p < 0.05.

In diary recordings, 7.6% (n = 18) of the adolescents recorded no headache episodes during the 3-week recording period. Of those who experienced headaches rated on the same 1-3 severity scale as in the questionnaire, 90.3% of the adolescents had low levels, while 8.3% and 1.4% of them experienced medium or high levels of headaches, respectively. While no association between gender and grade was found, a significantly higher proportion of students in the town (17.6%) had medium or high headache levels than those in the city (6.1%), χ2 (4) = 10.08, p < 0.01. The results of Wilcoxon test showed that adolescents reported significantly higher severity levels in the questionnaire than in diary recordings, Z = -5.76, p < 0.001.

Further analysis was carried out on the agreement between severity levels (recoded into a 1-3 scale: low, medium and high levels) in the questionnaire and average mean levels in the diary subdivided into 1-3 levels. The results showed that of adolescents with low severity levels of headache in the questionnaire, 95.2% also had the same levels in prospective diary recordings, while 4.8% had medium levels. For those who reported medium headache levels in the questionnaire, only 14.3% had the same levels of headaches in the diary, all the others had low levels. Of those who reported high headache levels in the questionnaire, only 2 out of 23 (8.7%) had the same level in diary recordings, and 17.4% had medium and 73.9% low levels.

#### Headache frequency

To compare number of headaches over a full month, the number of headache days for the 3-week period in diary recordings was converted (multiplied by 1.43) to individual numbers for a full 30 day period. In the questionnaire, 5% of students reported non-disease-related headaches occurring less than once a month, 35.1% 1-3 times a month, 36.5% 1-3 times a week, and 23.4% every day or almost every day. The corresponding percentages as reflected by diary recordings were: 9%, 6.8%, 42.3% and 41.9%, respectively. The results of Wilcoxon paired test showed that ratings of headache frequency were significantly (Z = -4.39, p < 0.001) higher in the diary than in the questionnaire format.

Whereas the association between ratings of the same 1-3 categories (less than once a month and 1-3 times a month collapsed) in the questionnaire and diary was significant, χ2 (4) = 12.44, p < 0.05, the agreement was poor. Out of the adolescents (n = 11) who reported headaches occurring less than once a month in the questionnaire, all of them reported higher frequency rates in the diary. For ratings of headache occurrence of 1-3 days per month in the questionnaire, 39% had the same level in diary recordings, while 18.2% had a lower frequency and 42.9% had higher rates. Similar findings were observed for headaches occurring every day or almost every day in questionnaire ratings, in which 38.5% of the adolescents also reported the same levels in diary recordings, but 14.6% and 46.9% had low or medium levels of headache frequency, respectively.

In the questionnaire, 10.3% of the adolescents reported headaches occurring every day or almost every day for at least 6 months defined as chronic headaches, 11.9% of the girls and 8.4% of the boys, a nonsignificant association.

#### Headache duration

In the questionnaire, 95.9% of all adolescents reported headaches up to 4 hours of duration, while in diary recordings about half of the sample (46.8%) reported such duration levels, here used as a cutoff for definition of shorter and longer episodes. About half of the students (47.9%), who reported a shorter duration of headache episodes in the questionnaire, also did so in the diary recordings. By contrast, of the 9 adolescents who reported longer headache episodes in the questionnaire, all had the same levels in the diary. Whereas the association between questionnaire and diary data for headaches up to 4 hours versus longer duration was significant, χ2 (2) = 14.06, p < 0.001, the overall agreement between the two information sources was low as reflected by a kappa of 0.11.

#### Intensity

On the 1-3 intensity scale for endorsement of mild, moderate and severe intensity levels in the questionnaire, 28.2% of the adolescents with nondisease-related headaches reported mild intensity levels, and for moderate and severe headaches, the figures were: 61.9% versus 9.9%, respectively.

#### Current headaches

Of the students in grades 6-9, 59.1% reported having current headaches at the assessment point with a grand mean on the 0-10 VAS intensity ratings of 1.91, SD = 2.39 (see Table 3). The results of two-way ANOVA and Student t-test showed nonsignificant mean differences for gender, grade and school location. Girls had somewhat higher VAS scores than boys, and students in the lowest grade had the highest scores descending up to grade 9 with all differences being nonsignificant.

#### Correlations between headache characteristics in the questionnaire and diary

The size and significance values for the various Spearman rank-order correlations are shown in Table 3. While ratings of headache severity (scale 1-5) in the questionnaire correlated positively with all other measures, the size of the correlations was consistently low. Headache frequency (scale 1-4) as reported in the questionnaire correlated negatively with the intensity measure (1-3 scale) in retrospect ratings and current headaches (VAS 0-10 scale) as well as with number of headache days as recorded in the diary, again with low correlation sizes. The experience of current headaches showed the highest and a positive correlation with number of headache days in the diary. Severity ratings in diary recordings correlated positively with duration of headache episodes, again with a low size. The latter measure showed the highest and a positive correlation with number of headache days as reflected in diary recordings.

## Discussion

In the present study of a convenience sample recruited from six different schools in one city and in one town, 237 adolescents in grades 6-9 and second year in high school (age range 12-18 years) participated in a survey of headaches and comparisons between two information methods and sources, that is retrospective questionnaire data vs. prospective recordings in a commonly used and standardized paper diary. The sample included adolescents who retrospectively reported headaches not related to disease in the questionnaire, and who also completed prospective diary recording during a 3-week period. Almost all of them had experienced headaches for at least one year, and about two-thirds had had more than ten previous episodes of headaches, the majority consisting of tension-type headaches typically observed in community populations.

Whereas numerous epidemiological surveys of community samples have reported estimates of subjects with no headache [4,5], sparse information exists on the proportion of children and adolescents who experience headaches related to various forms of disease. About one third of the adolescents (33%), who reported no headache during the last year or headaches only related to fever or infection or other disease, were excluded in our comparisons between questionnaire and diary data. In a Finnish epidemiological survey of unselected 12-year-old children in a community sample, about one third had no headaches, and 12% reported headaches associated with disease, most commonly infections in the head [35]. In a recent epidemiological survey of Norwegian school adolescents, 7.4% reported headaches only associated with illness such as flu or fever [Krogh, Larsson & Linde: Prevalence and disability of headache among Norwegian adolescents: a general population-based study, submitted for publication].

To the best of the authors’ knowledge, only a few similar epidemiological surveys have been conducted in which various types of paper diary recordings have been used to assess headaches in school-aged children in community samples [11,13,14]. Our study design and assessment methods have many similarities with the ones used in a previous Dutch study by van den Brink and collaborators [13]. In their study, 88% of invited school children aged 9-16 years (N = 186), who had participated earlier in a questionnaire survey and who experienced weekly headaches or more often, completed a 4-week diary using the same format, time points for daily recordings on the same severity scale as in the present study. Two percent of the students in the Dutch study did not report any headache in diary recordings, whereas in the present study 8% did so, but this included somewhat older students aged 12-18 years, who had experienced headaches of any frequency not related to disease during the last year.

Overall, our findings showed that adolescents overestimated headache severity and underestimated frequency as well as duration of headaches in retrospective questionnaire ratings as compared to prospective diary recordings. However, a closer look at the agreement between ratings in the two assessment methods showed that the concordance varied in regard to levels of headache on the various characteristics. Whereas almost all adolescents reported the same low levels of headache severity in the questionnaire as in the diary, for medium and high levels, only a small proportion (8.7-14.3%) of the adolescents reported the same levels in the diary, thus reflecting a strong overestimation of more severe headaches in retrospective questionnaire ratings. By contrast, for headache frequency, almost all adolescents who reported low frequency of headaches occurring less than once a week, reported higher levels in the diary. For shorter headache episodes (up to 4-5 hours) reported by almost all adolescents in the questionnaire, approximately half of them (52.1%) recorded a longer headache duration in diary ratings, while all of the nine adolescents who reported longer headache duration in the questionnaire also did the same in diary recordings. The differences in directions of estimates of headache characteristics obtained by the two assessment methods may depend on the following reasons. Adolescent overestimate of severity of headaches in retrospective recall might be due to an overall evaluation and experience of its affective and social impact in everyday life, and specific information asked on the occurrence and duration of headaches produce lower estimates due to memory bias.

Similar to our findings, in the survey by van den Brink and collaborators [13], the school children strongly overestimated intensity of headaches in questionnaire ratings as compared to diary recordings. While medians for headache frequency were the same for questionnaire and diary ratings, the children both under- and overestimated headache frequency. In contrast to the finding of the present study, duration of headaches was overestimated in the questionnaire as compared to diary recordings. However, in line with our results, agreement between the two assessment methods depended on duration level. Overall, the correlations between measures were also as low as in the present study reflecting high intra-individual variation in both studies of community samples.

In a previous study of a random subsample of school children aged 7 to 17 years by Laurell and collaborators [14], the agreement between retrospective questionnaire and interview information versus subsequent prospective 3-week recordings was low. As in the present study, estimates of headache frequency in questionnaires were substantially higher in diary recordings, both assessment methods also being identical to the ones used in the present study. Further, no significant correlation was found between mean intensity of the headache episodes in diaries and those reported in questionnaires and interviews.

In earlier clinical studies of children primarily suffering from migraine [20-22] which included selected children likely to suffer from more severe and complicated forms of migraine [36], questionnaires were found both to overestimate and underestimate frequency and intensity of headache as compared to prospective diary recordings using the same format as in the present study [20-22].

However, our results on headache frequency contrast with findings in a recent clinical study of adolescents with frequent migraine, in which their recall of headache frequency on a questionnaire showed high agreement with recordings in an internet-based diary for both 30-day and 90-day intervals [19]. For a longer assessment period of 2-7 months, Metsähonkala and collaborators [18] noted that duration of migraine among 11-13-year olds was significantly longer when recorded prospectively in a diary than in interviews and memories of retrospective attack duration. In a clinical study of chronic pain including headaches, children and adolescents reported lower pain intensity levels in a diary compared to a questionnaire [17].

Some limitations of the present study need to be acknowledged. It is based on a convenience sample where teachers selected the participating classes. Headaches among students were assessed for a limited 3-week period in the middle of the spring semester when school work load and stress are generally high, which might have produced higher estimates of headache occurrence. While the comparisons between the retrospective and prospective assessment methods covered adjacent time periods, they were also somewhat different in time, which likely contributed to some of the discrepancies in adolescent ratings of headaches. Students were asked to record their headache complaints four times a day, however, it is unknown to what degree they adhered to the predetermined times or relied on recall. The completion rate also declined somewhat over the 3-week period possibly due to tiredness in recording, which is likely to reduce the reliability and validity of the headache data [15].

The strengths of the study are the relatively large sample including adolescents in a school population from six different public schools in a city and a smaller town in the same county that also served rural areas. The sample represented age groups in which recurrent headaches of different severity levels are common. Disease-related headaches reported by the adolescents in the questionnaire were excluded, restricting our findings to headaches not related to self-report of common somatic diseases in a community population, such as cold and fever. The response rates in completion of questionnaires and diary recordings were also acceptable. In our previous study of headache prevalence and characteristics elicited through diary recordings [15], the estimates were strikingly higher, possibly due to the inclusion of low intensity headaches commonly ignored in previous epidemiological surveys.

## Conclusions

Although retrospect information is commonly used in clinical and health care interviews with adolescents and in large-scale research surveys where questionnaires are administered, it is clearly affected by recall bias and error that likely produce overestimates of headache characteristics. The use of prospective diary recordings will serve as an important compliment to obtain more valid and reliable information on recurrent headaches in children and adolescents in various settings. Given that many adolescents today have access to computers and mobile phones, the development of internet-based concurrent reports in the assessment of pain and headaches in these age groups are likely to be more practical and economical for use not only in large-scale epidemiological surveys, but also in regular health care services.

## Competing interests

The authors declare that they have no competing interests.

## Authors’ contributions

BL conceived the design of the study, performed the statistical analyses and wrote up drafts of the manuscript. ÅF participated in the planning of the study, collected parts of the data and read earlier drafts of the manuscript. Both authors read and approved the final version of the manuscript.
